# The Mechanism Study of Common Flavonoids on Antiglioma Based on Network Pharmacology and Molecular Docking

**DOI:** 10.1155/2022/2198722

**Published:** 2022-01-31

**Authors:** Taiping Li, Yong Xiao, Zhen Wang, Hong Xiao, Hongyi Liu

**Affiliations:** ^1^Department of Neuro-Psychiatric Institute, The Affiliated Nanjing Brain Hospital of Nanjing Medical University, Nanjing, China; ^2^Department of Neurosurgery, The Affiliated Nanjing Brain Hospital of Nanjing Medical University, Nanjing, China

## Abstract

**Background:**

Glioma is the most common primary intracranial tumor in adult patients. Among them, glioblastoma is a highly malignant one with a poor prognosis. Flavonoids are a class of phenolic compounds widely distributed in plants and have many biological functions, such as anti-inflammatory, antioxidant, antiaging, and anticancer. Nowadays, flavonoids have been applied to the therapy of glioma; however, the molecular mechanism underlying the therapeutic effects has not been fully elaborated. This study was carried out to explore the mechanism of selected active flavonoid compounds in treating glioma using network pharmacology and molecular docking approaches.

**Methods:**

Active ingredients and associated targets of flavonoids were acquired by using the Traditional Chinese Medicine Database and Analysis Platform (TCMSP) and Swiss TargetPrediction platform. Genes related to glioma were obtained from the GeneCards and DisGeNET databases. The intersection targets between flavonoid targets and glioma-related genes were used to construct protein-protein interaction (PPI) network via the STRING database, and the results were analyzed by Cytoscape software. Gene Ontology (GO) and Kyoto Encyclopedia of Genes and Genomes (KEGG) pathway enrichment analyses were performed and displayed by utilizing the Metascape portal and clusterProfiler R package. Molecular docking was carried out by iGEMDOCK and SwissDock, and the results were visually displayed by UCSF Chimera software.

**Results:**

Eighty-four active flavonoid compounds and 258 targets overlapped between flavonoid targets and glioma-related genes were achieved. PPI network revealed potential therapeutic targets, such as AKT1, EGFR, VEGFA, MAPK3, and CASP3, based on their node degree. GO and KEGG analyses showed that core targets were mainly enriched in the PI3K-Akt signaling pathway. Molecular docking simulation indicated that potential glioma-related targets-MAPK1 and HSP90AA1 were bounded more firmly with epigallocatechin-3-gallate (EGCG) than with quercetin.

**Conclusions:**

The findings of this study indicated that selected active flavonoid compounds might play therapeutic roles in glioma mainly through the PI3K-Akt signaling pathway. Moreover, EGCG had the potential antiglioma activity by targeting MAPK1 and HSP90AA1.

## 1. Introduction

Glioma, originated from the neuroepithelium, accounts for 40%∼50% of brain tumors and is one of the most common primary intracranial tumors; among them, glioblastoma is a highly malignant one with poor clinical outcome [1]. Extensive studies have shown that flavonoids have a good therapeutic effect on glioma [2–4]; however, the underlying therapeutic molecular mechanisms of flavonoids on glioma are not stated clearly. Hence, a systematic exploration of the molecular mechanisms of flavonoids on glioma is critical. Network pharmacology is an emerging interdisciplinary discipline and has been applied to comprehensively analyze the functional mechanisms of traditional Chinese medicine [5]. It is also used to reveal the active ingredients of natural medicine treating glioma [6].

**Figure 1:**
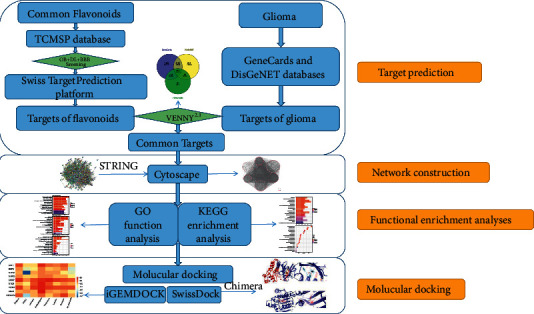
Study flowchart of the molecular mechanism of flavonoids in treating glioma.

In this study, we tried to systematically identify the molecular mechanisms of flavonoids' antiglioma effects based on findings from network pharmacology and molecular docking. The flowchart of this study is shown in Figure 1. Our work portrays the ground view of antiglioma molecular mechanisms of flavonoids, which provided active compounds and therapy targets curing glioma.

## 2. Materials and Methods

### 2.1. Screening Active Ingredients and Predicting Related Targets

TCMSP (https://tcmsp-e.com/) is a unique systematic pharmacology platform for Chinese herbal medicines and is characterized by exploring relationships between compounds, targets, and diseases. TCMSP has been utilized to screen active flavonoid ingredients [7]. Flavonoids are categorized according to their molecular structures into flavones, flavonols, isoflavones, chalcones, flavones, and anthocyanidins [4], as shown in Table 1. Key parameters were taken into account, such as oral bioavailability (OB), drug-likeness (DL), and blood-brain barrier (BBB). OB is a measurement of the proportion of drugs entering the blood circulation. DL indicates the potential of a compound to be developed into a therapeutic drug with respect to its physical and chemical properties. BBB impedes drug distribution between blood and brain [8], which is a very important parameter in the treatment of glioma. In general, one compound is considered permeable across the BBB when its BBB permeability is larger than −0.30. Active flavonoids were acquired by the following criteria: OB ≥ 30%, DL ≥ 0.18, and BBB ≥ −0.3 [9, 10]. Active compounds of flavonoids reported publicly in recent five years were also taken into analyses from 1^st^ January 2016 to 30^th^ May 2021. The active flavonoids were confirmed by PubChem (https://pubchem.ncbi.nlm.nih.gov/). The structures of compounds saved in SDF format were used to predict potential targets of active molecules by using Swiss TargetPrediction platform (http://www.swisstargetprediction.ch/), and the species were set as “Homo sapiens” and the probability was set larger than 0. All the targets from UniProt database (https://www.uniprot.org/) were in standardized format.

### 2.2. Determination of Glioma-Related Targets and Common Targets with Active Components

Glioma-related targets were retrieved by using the GeneCards (https://www.genecards.org/) and DisGeNET (https://www.disgenet.org/home/) databases with the keyword “glioma.” Flavonoid targets and glioma-related genes were imported into Venny 2.1 (https://bioinfogp.cnb.csic.es/tools/venny/) to acquire common targets as the potential targets for further analyses.

### 2.3. Network Construction of Common Targets

A protein-protein interaction (PPI) network was constructed using the STRING platform (version 11.5, https://string-db.org/), and “*Homo sapiens*” and *Medium Confidence* (0.4) were set. All the information derived from the STRING database was then imported into Cytoscape software (version 3.8.2) for visual display.

### 2.4. GO Function and KEGG Pathway Enrichment Analyses

GO is wildly used to study gene functions, including the biological process (BP), molecular function (MF), and cell component (CC) [11]. KEGG (http://www.kegg.jp/) is an integrated database of genomic, chemical, and system functional information and is extensively used to capture significantly enriched biological pathways [12]. The common targets of flavonoid-glioma were imported into the Metascape portal (http://metascape.org/gp/index.html/) for enrichment analysis. The top 20 GO and KEGG pathway enrichment analysis results were visualized by clusterProfiler R package as the histogram or bubble graph with *p* < 0.01 [13].

### 2.5. Molecular Docking

The most potential pathway and its related genes of flavonoids treating glioma were obtained. Then, these targets with the most promising ingredients were reconfirmed by using molecular docking. Crystal structures of related proteins were obtained from the RCSB Protein Data Bank (PDB, https://www.rcsb.org/) with high resolution and score, water was removed, while hydrogens were added by MGLTools software (version 1.5.6). Verified compounds in.mol2 format were acquired from the TCM@Taiwan database (https://tcm.cmu.edu.tw/). Molecular docking was carried out using iGEMDOCK software (version 2.1) with default parameters. We selected the most potential proteins which had the lowest energy and determined their docking ligands using the SwissDock platform (http://www.swissdock.ch/docking/). The results were visually displayed by UCSF Chimera software (version 1.15).

## 3. Results

### 3.1. Active Ingredients of Flavonoid

According to the TCMSP database, 55 compounds were screened out with the thresholds of OB ≥ 30%, DL ≥ 0.18, and BBB ≥ −0.3 (Table 2). Twenty-nine compounds were ruled out due to the aforementioned screening conditions, but they have been reported to have antiglioma properties in previous studies [2, 3, 14–65]. We added them into our study to decipher the whole view of flavonoids' antiglioma molecular mechanism. As a result, a total of 84 active compounds were selected for further analysis. Quercetin, epigallocatechin-3-gallate, isoliquiritigenin, genistein, apigenin, kaempferol, and luteolin had 154, 140, 124, 97, 80, 63, and 57 targets, respectively. It revealed that these seven flavonoids probably played significant roles in curing glioma (Table 3).

### 3.2. Overlapping Common Targets of Flavonoid-Glioma

**Figure 2:**
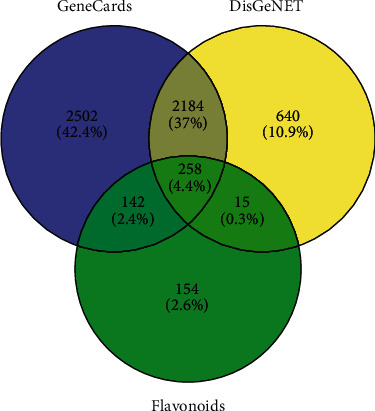
Overlapping target genes between glioma and flavonoids.

After exclusion of duplicated data, 5086 and 3097 glioma-related targets were identified from GeneCards and DisGeNET databases, respectively, and 569 candidate targets of active flavonoids were integrated from Swiss TargetPrediction. Two hundred and fifty-eight intersection targets were obtained among these three gene sets and were used for further analysis (Figure 2).

### 3.3. Common Targets Network Construction

**Figure 3:**
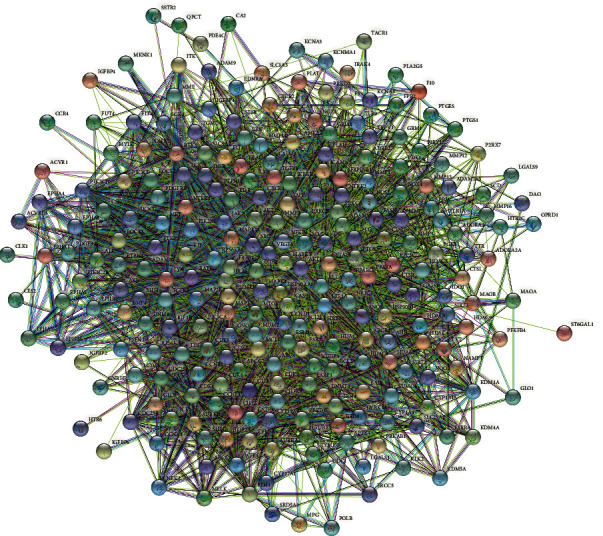
PPI network of potential targets generated by STRING (258 nodes, 4407 edges).

**Figure 4:**
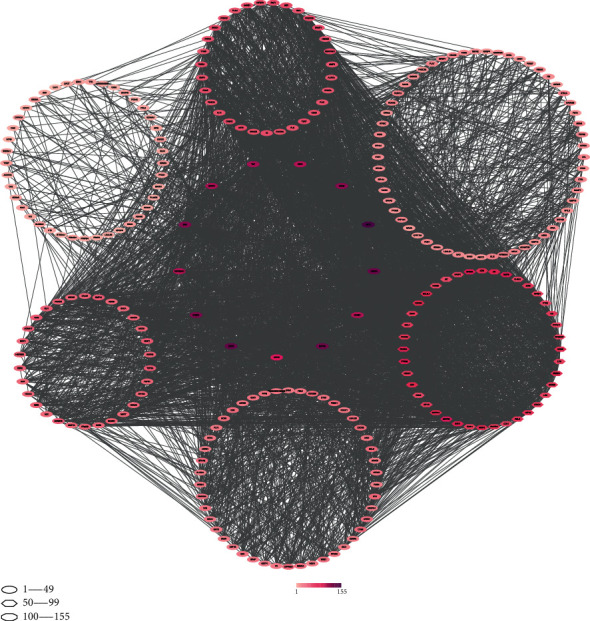
PPI network of common targets of flavonoids treating glioma. The node color was in proportion to the number of interacted nodes, and the more nodes the node linked, the deeper color it showed.

These 258 putative gene targets correlated with glioma were analyzed using the STRING database. A total of 258 nodes and 4407 edges were embodied with the average node degree 34.2. These results were imported into Cytoscape software (version 3.8.2) for further analysis. The network is shown in Figure 3. The node color reflected the number of interacted nodes, and the more nodes to one node linked with, the deeper colored it became, as shown in Figure 4.

The potential targets were AKT1, EGFR, VEGFA, MAPK3, CASP3, SRC, HRAS, TNF, MAPK1, CCND1, ESR1, HSP90AA1, and MTOR as their degrees were above 100 (Table 4). Node degrees were counted by Cytoscape. The greater a node degree is, the more important biological functions the node has in the PPI network (Table 5).

### 3.4. GO and KEGG Pathway Enrichment Analyses

**Figure 5:**
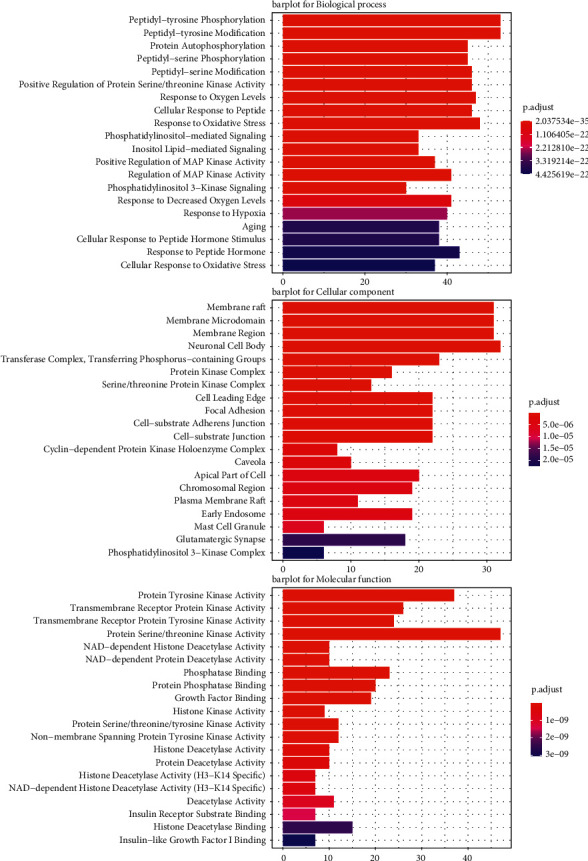
GO enrichment analysis of biological process (BP) terms, cellular component (CC) terms, and molecular function (MF) terms. The color of the bar is displayed in a gradient from red to blue according to the ascending order of the *P* adjust, while the length of the bar is arranged according to the ascending order of the number of gene counts.

GO terms were enriched by the Metascape platform. The results showed that BP terms enriched in glioma-flavonoids overlapping targets mainly included peptidyl-tyrosine phosphorylation and modification, response to oxidative stress and oxygen levels. The top five enriched CC terms were membrane raft, membrane microdomain, membrane region, neuronal cell body, and transferase complex, transferring phosphorus-containing groups. MF terms displayed the intersection genes that were mainly enriched in protein tyrosine kinase activity, protein serine/threonine kinase activity, transmembrane receptor protein kinase activity, transmembrane receptor protein tyrosine kinase activity, and phosphatase binding (Figure 5).

**Figure 6:**
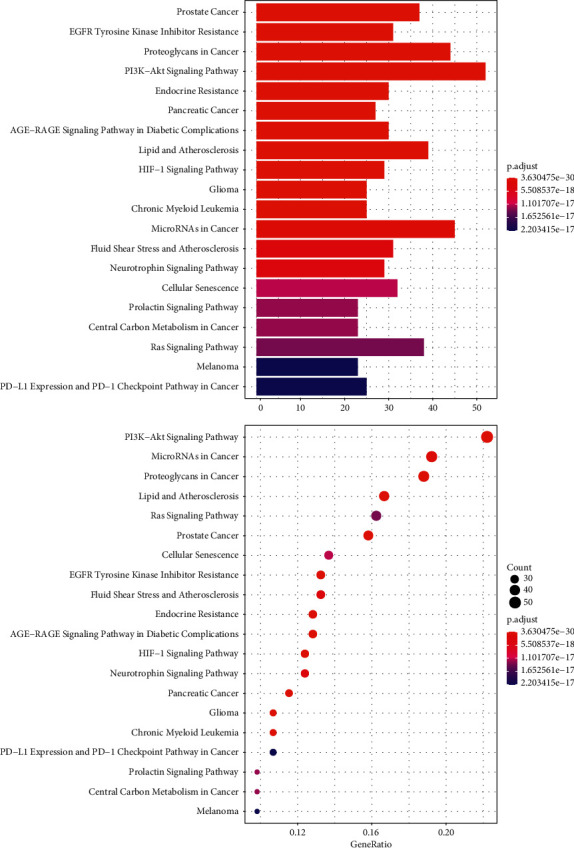
KEGG pathway analysis of potential targets of flavonoids on glioma-related proteins visualized by the clusterProfiler. The color of the bar is displayed in a gradient from red to blue according to the *P* adjust, while the sizes of dots are arranged according to the ascending order of the number of gene counts.

KEGG pathway enrichment analysis of the 258 intersection gene targets was carried out by Metascape. The main pathways among these genes included PI3K-Akt, Ras, HIF-1, and Neurotrophin signaling pathways (Figure 6).

### 3.5. Molecular Docking

**Figure 7:**
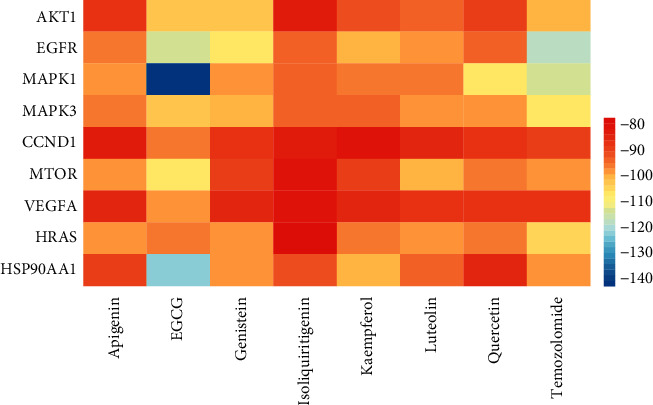
Heatmap of molecular docking. Temozolomide was taken as the control. The red color represents a high docking score, and blue represents a low docking score. The lowest value indicates the most stable conformation.

**Figure 8:**
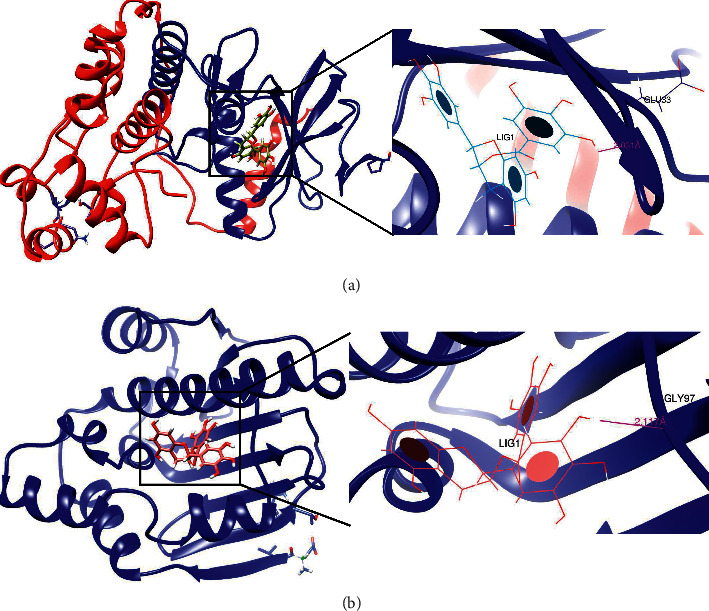
The binding modes of EGCG with MAPK1 (a) and HSP90AA1 (b). Left panel: this area showed the best combination pocket of EGCG with MAPK1 and HSP90AA1 proteins. EGCG and relevant residues were presented in stick representation. Right panel: this region showed the hydrogen bonding with Glu33 in MAPK1 protein and Gly97 in HSP90AA1 protein. The distance of hydrogen bond interaction was colored in pink, and the structure of EGCG was shown in circle and disk shape.

The result of the KEGG pathway enrichment analysis indicated that the PI3K-Akt signaling pathway was the main pathway through which flavonoids affected the glioma. Among the potential targets, AKT1, EGFR, MAPK1, MAPK3, CCND1, MTOR, VEGFA, HRAS, and HSP90AA1 were enriched in the PI3K-Akt signaling pathway. We selected seven potential active molecules, including quercetin, epigallocatechin-3-gallate, isoliquiritigenin, genistein, apigenin, kaempferol, and luteolin, to dock with nine target proteins, and chose temozolomide as the control. Lower binding energy indicates a stabler conformation. We used the quantitative value of fitness to evaluate the binding level. Fitness is the total energy of a predicted pose in the binding site. The empirical scoring function of iGEMDOCK is estimated as follows: Fitness = vdW + Hbond + Elec. The vdW term is van der Waal energy; Hbond and Elect terms are hydrogen bonding energy and electrostatic energy, respectively [66]. The results were visually displayed with a heatmap (Figure 7). It was interesting to note that epigallocatechin-3-gallate (EGCG) had a good bonding ability to most target proteins enriched in the PI3K-Akt signaling pathway, while the opposite pattern was observed for isoliquiritigenin. Experimental studies show that quercetin could induce autophagy and apoptosis in human neuroglioma cells through the PI3K-Akt signaling pathway [67]. AKT1, MTOR, CCND1, and EGFR are closely associated with autophagy and apoptosis in glioma [68–70]. Our findings obviously showed that EGCG had a better docking score to these proteins than quercetin. For further research, quercetin and EGCG were selected in this study to dock with MAPK1 and HSP90AA1 target proteins individually due to their relatively lower energy value in the molecular docking. The results of SwissDock revealed that the estimated Gibbs free energies (ΔG) of best binding modes of EGCG with two targeting proteins were −9.27 kcal/mol and −8.53 kcal/mol, respectively, while the binding energies of quercetin with two targeting proteins were −8.23 kcal/mol and −7.95 kcal/mol, separately. In addition, EGCG had one backbone hydrogen bond (HB) interacting with Glu33 of MAPK1, and the distance was 2.031 Å. One backbone HB bounded with Gly97 of HSP90AA1 with a distance of 2.117 Å. The results displayed by UCSF Chimera software were shown in Figure 8.

## 4. Discussion

In recent years, flavonoids are widely used for antiglioma treatment. The mechanisms of flavonoids are very complex because they have multiple potential targets and active components. Network pharmacology together with bioinformatics has superiority in the systematic elucidation of the mechanism of TCM at the molecular level and representation of interactions between active compounds, potential targets, and various pathways.

In our study, potential targets of active components analysis revealed that quercetin, EGCG, isoliquiritigenin, genistein, apigenin, kaempferol, and luteolin interacted with multiple targets in the network. These findings showed that they may play important roles in the treatment of glioma. As reported, these seven active flavonoid compounds have distinct ways of treating glioma. Quercetin, a flavonol, had the most potential targets in this study. It plays antiglioma effects by inducing cell apoptosis [17], inhibiting proliferation and migration [14], and modulating the inflammatory process [71]. Moreover, quercetin could affect human glioma cells through the PI3K-Akt signaling pathway [72]. EGCG is a polyphenol flavonoid, which is generally distributed in green tea and has shown great properties in cancer prevention due to its safety, low cost, and excellent bioavailability [73]. EGCG in high doses (>40 *μ*mol/L) could suppress cancer cells by inducing apoptosis and by inhibiting autophagic processes [74] and regulate apoptosis-related and autophagy-related proteins (caspase3, caspase 9, Bax, LC3B II, and Beclin) [75]. MTOR is a key regulator of autophagy, EGCG may enhance the phosphorylation of eNOS and mTOR via the activation of the PI3K-Akt pathway [76]. Furthermore, it has the effects of antiglioma through inhibiting proliferation and decreasing invasion of glioma cells [77]. Isoliquiritigenin, isolated from licorice, has been found to be a potent stimulator of cell differentiation and has potential application for treating human brain glioma by inhibiting proliferation and blocking angiogenic through Notch1 and Akt signaling pathway, respectively [22, 23]. Genistein, an isoflavone in legumes and some herbal medicines, suppresses the expression of matrix metalloproteinase 2 (MMP-2) and vascular endothelial growth factor (VEGF) to serve antigiloma role [78]. Genistein sensitizes glioblastoma cells to carbon ions through inhibiting DNA-PKcs phosphorylation and subsequently repressing the nonhomologous end-joining and delaying the homologous recombination repair pathways [24]. Apigenin, a flavone, has been shown to take part in restoring the immune system and weakening the self-renewal and invasiveness capacity of glioblastoma stem-like cells (GSCs) [25, 29]. It was reported to inhibit the expression of STAT3, AKT, and MAPK in the GSCs [26]. Kaempferol has also been demonstrated to possess good antiglioma effects by inducing reactive oxygen species (ROS) and subsequently leads to autophagy and cell death [30, 79]. Luteolin is a flavone and has an inhibitory effect on downstream signal molecules activated by EGFR, particularly the Akt and MAPK signal pathways [33, 80]. It induces a lethal endoplasmic reticulum stress response and mitochondrial dysfunction in glioblastoma cells by increasing intracellular ROS levels [31].

Immune factors have been considered as a significant factor contributing to the development and progression of glioma [81]. In the PPI network, most potential targets were closely related to immunity, including AKT1, TNF, EGFR, VEGFA, MAPK1, MAPK3, CASP3, SRC, HRAS, CCND1, ESR1, HSP90AA1, and MTOR [82–87]. And these proteins were regarded as core proteins in our study and might play important roles in the therapeutic effect of flavonoids on glioma. Recent studies have shown that luteolin decreased the expression of immune-related genes including MMP9, MAPK1, HSP90AA1, CASP3, ALB, EGFR, SRC, HRAS, and ESR1. And among these genes, MMP9, MAPK1, HSP90AA1, EGFR, SRC, and HRAS are confirmed *in vivo* at the protein and mRNA levels [88].

To further indicate the potential mechanism of flavonoids in treating glioma, KEGG analysis discovered that PI3K-Akt was the main signaling pathway. It is a classic signal transduction pathway involved in cell proliferation, apoptosis, migration, invasion, and angiogenesis in glioma and plays an important role in the occurrence and development of glioma [89]. The result of molecular docking showed that EGCG had good bonding with MAPK1 and HSP90AA1 in the PI3K-Akt signaling pathway. Relevant studies confirmed that EGCG induces apoptosis, inhibits proliferation, and decreases invasion of glioma cells via the MAPK pathway *in vivo* [77]. Kim et al. also found that EGCG induced the expression of MAPK1 in glioma cells [90]. Heat Shock Protein 90 can promote oncogenesis since it interacts and supports numerous proteins and is essential for malignant transformation and progression. However, the HSP90AA1 gene is not altered in a major of tumors according to the Cancer Genome Atlas (TCGA) [91]. To evaluate its role in the treatment of glioma, downregulation of HSP90AA1-IT1 (HSP90AA1 intronic transcript (1) was done, which could significantly suppress cell viability, proliferation, EMT, invasion, and migration of glioma [92]. Thus, there might be a correlation between HSP90AA1 and glioma; however, there is no report about EGCG curing glioma via targeting HSP90AA1.

Although there is an abundance of information and the analysis process is complex, some useful and credible conclusions have been drawn. Due to limitations of compounds screening and accuracy of target prediction, the results obtained in this study are general, and *in vitro* and *in vivo* experiments are needed for verification. In short, our study portrayed the ground view of flavonoids in the treatment of glioma.

## 5. Conclusions

This study elaborated the mechanisms of active flavonoids on antiglioma using network pharmacology and molecular docking by constructing a compound‒target‒pathway network. Active components have particular advantages in curing glioma by targeting MAPK1, MAPK3, EGFR, MTOR, AKT1, VEGFA, CCND1, HSP90AA1, and HRAS. In addition, EGCG can target HSP90AA1 and MAPK1 via the PI3K-Akt signaling pathway. These findings offered a research foundation for further investigation of flavonoids on antiglioma.

## Figures and Tables

**Table 1:** Main structure of flavonoids with their representative compounds.

Types	Main structure	Representative compounds
Flavones	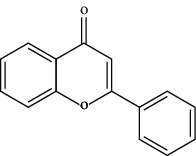	Apigenin, luteolin
Flavonols	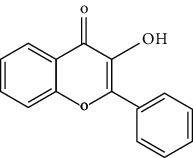	Quercetin, myricetin
Isoflavones	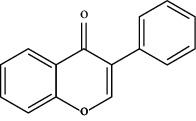	Genistein, daidzein
Chalcones	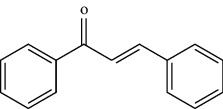	Isoliquiritigenin, corylifolinin
Flavanones	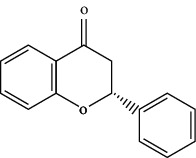	Naringenin, hesperidin
Anthocyanidins	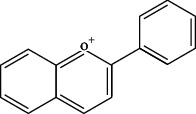	Cyanidin, pelargonidin

**Table 2:** Basic information on the main active flavonoids.

No.	Molecule ID	Molecule name	Related targets	OB (%)	BBB	DL
1	MOL000173	Wogonin	45	30.68	0.04	0.23
2	MOL003896	7-Methoxy-2-methyl isoflavone	43	42.56	0.56	0.20
3	MOL000392	Formononetin	39	69.67	0.02	0.21
4	MOL002714	Baicalein	37	33.52	−0.05	0.21
5	MOL005828	Nobiletin	35	61.67	−0.08	0.52
6	MOL001876	6-Methoxyflavone	34	34.56	0.49	0.18
7	MOL007879	Tetramethoxyluteolin	32	43.68	0.09	0.37
8	MOL000497	Licochalcone A	32	40.79	−0.21	0.29
9	MOL004957	HMO	27	38.37	0.25	0.21
10	MOL004835	Glypallichalcone	27	61.60	0.23	0.19
11	MOL013277	Isosinensetin	27	51.15	0.03	0.44
12	MOL002928	Oroxylin A	26	41.37	0.13	0.23
13	MOL004828	Glepidotin A	26	44.72	0.06	0.35
14	MOL001689	Acacetin	26	34.97	−0.05	0.24
15	MOL008206	Moslosooflavone	25	44.09	0.54	0.25
16	MOL004991	7-Acetoxy-2-methylisoflavone	25	38.92	0.16	0.26
17	MOL008239	Quercetin tetramethyl(3′,4′,5,7) ether	23	31.57	0.36	0.41
18	MOL005229	Artemetin	23	49.55	−0.09	0.48
19	MOL008400	Glycitein	23	50.48	−0.29	0.24
20	MOL012266	Rivularin	22	37.94	−0.13	0.37
21	MOL000507	Psi-Baptigenin	22	70.12	−0.27	0.31
22	MOL001803	Sinensetin	21	50.56	0.04	0.45
23	MOL000552	5,2′-Dihydroxy-6,7,8-trimethoxyflavone	21	31.71	0	0.35
24	MOL002927	Skullcapflavone II	21	69.51	−0.07	0.44
25	MOL011078	3′,7-dihydroxy-4′-methoxy-isoflavone	21	50.70	−0.09	0.24
26	MOL003758	Iristectorigenin (9CI)	21	71.55	−0.16	0.34
27	MOL003656	Lupiwighteone	21	51.64	−0.23	0.37
28	MOL012101	Mosloflavone	19	34.04	0.29	0.26
29	MOL002563	Galangin	19	45.55	−0.09	0.21
30	MOL004883	Licoisoflavone	19	41.61	−0.27	0.42
31	MOL005012	Licoagroisoflavone	18	57.28	0.09	0.49
32	MOL002915	Salvigenin	18	49.07	−0.03	0.33
33	MOL004848	Licochalcone G	17	49.25	−0.04	0.32
34	MOL002917	5,2′,6′-Trihydroxy-7,8-dimethoxyflavone	17	45.05	−0.11	0.33
35	MOL004884	Licoisoflavone B	17	38.93	−0.18	0.55
36	MOL004564	Kaempferid	17	73.41	−0.21	0.27
37	MOL005321	Frutinone A	16	65.90	0.46	0.34
38	MOL013279	5,7,4′-Trimethylapigenin	16	39.83	0.12	0.30
39	MOL002235	Eupatin	16	50.80	−0.26	0.41
40	MOL012108	Negletein	15	41.16	0.13	0.23
41	MOL008127	Ermanin	15	58.95	0.07	0.30
42	MOL005573	Genkwanin	14	37.13	−0.24	0.24
43	MOL005849	Didymin	13	38.55	−0.07	0.24
44	MOL000239	Jaranol	13	50.83	−0.22	0.29
45	MOL004598	3,5,6,7-tetramethoxy-2-(3,4,5-trimethoxyphenyl)chromone	12	31.97	0.08	0.59
46	MOL005842	Pectolinarigenin	12	41.17	−0.09	0.30
47	MOL000525	Norwogonin	12	39.40	−0.17	0.21
48	MOL004114	3,2',4′,6′-Tetrahydroxy-4,3′-dimethoxy chalcone	11	52.69	−0.15	0.28
49	MOL002341	Hesperetin	9	70.31	−0.25	0.27
50	MOL006331	4′,5-Dihydroxyflavone	8	48.55	−0.03	0.19
51	MOL002398	Karanjin	5	69.56	0.62	0.34
52	MOL000242	7-O-Methyleriodictyol	5	56.56	−0.21	0.27
53	MOL002913	Dihydrobaicalin_qt	4	40.04	0.18	0.21
54	MOL002908	5,8,2′-Trihydroxy-7-methoxyflavone	NA	37.01	−0.07	0.27
55	MOL002719	6-Hydroxynaringenin	NA	33.23	−0.27	0.24

NA: not available.

**Table 3:** Active flavonoids in the treatment of glioma reported in PubMed in recent five years.

No.	Molecule ID	Molecule name	Related targets	Annotation
1	MOL000098	Quercetin	154	[14–19]
2	MOL006821	Epigallocatechin-3-gallate	140	[20, 21]
3	MOL001789	Isoliquiritigenin	124	[22, 23]
4	MOL000481	Genistein	97	[24]
5	MOL000008	Apigenin	80	[25–29]
6	MOL000422	Kaempferol	63	[30]
7	MOL000006	Luteolin	57	[31–35]
8	MOL013179	Fisetin	46	[36]
9	MOL002008	Myricetin	38	[37, 38]
10	MOL005734	Eupatilin	31	[39]
11	MOL005814	Tangeretin	29	[40, 41]
12	MOL000417	Calycosin	22	[42]
13	MOL002560	Chrysin	19	[43, 44]
14	MOL002083	Tricin	18	[45]
15	MOL009297	Jaceosidin	16	[46]
16	MOL005811	Hepta-3	14	[47]
17	MOL000492	(+)-Catechin	11	[2]
18	MOL002881	Diosmetin	10	[3]
19	MOL005093	Diosmin	10	[48]
20	MOL005190	Eriodictyol	9	[49]
21	MOL013374	Ampelopsin	7	[50]
22	MOL004925	Vitexin	6	[51, 52]
23	MOL002931	Scutellarin	6	[53]
24	MOL005812	Naringin	5	[54, 55]
25	MOL007450	Silybin	2	[43, 56, 57]
26	MOL001790	Linarin	1	[58, 59]
27	MOL004425	Icariin	1	[60]
28	MOL002037	Amentoflavone	NA	[61–64]
29	MOL007285	Procyanidol C1	NA	[65]

NA: not available.

**Table 4:** Potential active targets of flavonoids.

No.	Uniprot ID	Target name	Protein name	Degree
1	P31749	AKT1	RAC-alpha serine/threonine-protein kinase	155
2	P00533	EGFR	Epidermal growth factor receptor	139
3	P15692	VEGFA	Vascular endothelial growth factor A	136
4	P27361	MAPK3	Mitogen-activated protein kinase 3	134
5	P42574	CASP3	Caspase-3	128
6	P12931	SRC	Protooncogene tyrosine-protein kinase Src	126
7	P01112	HRAS	GTPase HRas	124
8	P01375	TNF	Tumor necrosis factor	115
9	P28482	MAPK1	Mitogen-activated protein kinase 1	115
10	P24385	CCND1	G1/S-specific cyclin D1	112
11	P03372	ESR1	Estrogen receptor	112
12	P07900	HSP90AA1	Heat Shock Protein HSP 90-alpha	111
13	P42345	MTOR	Serine/threonine-protein kinase mTOR	104

**Table 5:** Docking score of targets with seven active ingredients (kcal/mol).

Target name	PDB ID	Apigenin	EGCG	Genistein	Isoliquiritigenin	Kaempferol	Luteolin	Quercetin	Temozolomide
AKT1	6S9W	−87.88	−104.05	−103.38	−84.38	−91.79	−94.33	−90.26	−101.62
EGFR	7AEM	−96.74	−114.52	−106.53	−93.64	−100.48	−98.17	−95.28	−117.90
MAPK1	6RFP	−97.85	−143.55	−98.34	−94.73	−96.12	−95.77	−106.79	−113.58
MAPK3	6GES	−97.51	−102.29	−101.62	−94.60	−93.60	−99.52	−98.23	−107.29
CCND1	3AY5	−83.32	−96.44	−87.54	−83.87	−80.69	−86.00	−87.49	−90.09
MTOR	7JWE	−99.64	−107.48	−90.22	−82.14	−89.54	−100.05	−96.34	−97.78
VEGFA	6D3O	−84.52	−98.62	−85.47	−80.56	−85.32	−87.91	−86.79	−86.84
HRAS	4XVR	−98.48	−97.18	−98.35	−77.91	−96.60	−99.33	−96.30	−104.24
HSP90AA1	4BQG	−90.22	−123.19	−98.78	−92.72	−100.26	−94.73	−84.84	−98.88

## Data Availability

The data used to support the findings of this study are available from the corresponding author upon request.

## Abbreviations

BBB: Blood-brain barrier
BP: Biological process
CC: Cell component
DL: Drug-likeness
EGCG: Epigallocatechin-3-gallate
EMT: Epithelial-mesenchymal transition
eNOS: Endothelial nitric oxide synthase
GO: Gene Ontology
GSCs: Glioblastoma stem-like cells
HB: Hydrogen bond
HIF-1: Hypoxia-inducible factor-1
KEGG: Kyoto Encyclopedia of Genes and Genomes
MF: Molecular function
MMP-2: Matrix metalloproteinase 2
OB: Oral bioavailability
PDB: Protein data bank
PI3K-Akt: Phosphatidylinositol 3-kinase-protein kinase B
PPI: Protein-protein interaction
ROS: Reactive oxygen species
SDF: Structure-data file
STAT3: Signal transducer and activator of transcription 3
TCM: Traditional Chinese medicine
TCMSP: Traditional Chinese medicine database and analysis platform
ΔG: Gibbs free energy.
